# Studies of Fractal Microstructure in Nanocarbon Polymer Composites

**DOI:** 10.3390/polym16101354

**Published:** 2024-05-10

**Authors:** Igor Artyukov, Stefano Bellucci, Vladimir Kolesov, Vadim Levin, Egor Morokov, Maxim Polikarpov, Yulia Petronyuk

**Affiliations:** 1 X-ray Optics Laboratory, Lebedev Physical Institute, Russian Academy of Sciences, 119991 Moscow, Russia; iart@mail.ru; 2 INFN-Laboratori Nazionali di Frascati, 00044 Frascati, Italy; stefano.bellucci@lnf.infn.it; 3 Kotel’nikov Institute of Radio Engineering and Electronics, Russian Academy of Sciences, 125009 Moscow, Russia; kvv@cplire.ru; 4 Laboratory of Acoustic Microscopy, Emanuel Institute of Biochemical Physics, Russian Academy of Sciences, 119334 Moscow, Russia; levin1943@gmail.com (V.L.); es_morokov@yahoo.com (E.M.); 5 Department of Physics and Mathematics, Pirogov Russian National Research Medical University, 117997 Moscow, Russia; 6 European Molecular Biology Laboratory, Hamburg Unit c/o DESY, 22607 Hamburg, Germany; polikarpov.maxim@mail.ru; 7 Scientific and Technological Center of Unique Instrumentation, Russian Academy of Sciences, 117342 Moscow, Russia

**Keywords:** nanocomposite, synchrotron X-ray imaging, acoustic microscopy, exfoliated graphite, carbon platelets, CNT agglomeration, phase contrast, X-ray tomography, synchrotron radiation

## Abstract

The in situ study of fractal microstructure in nanocarbon polymers is an actual task for their application and for the improvement in their functional properties. This article presents a visualization of the bulk structural features of the composites using pulsed acoustic microscopy and synchrotron X-ray microtomography. This article presents details of fractal structure formation using carbon particles of different sizes and shapes—exfoliated graphite, carbon platelets and nanotubes. Individual structural elements of the composite, i.e., conglomerations of the particles in the air capsule as well as their distribution in the composite volume, were observed at the micro- and nanoscale. We have considered the influence of particle architecture on the fractal formation and elastic properties of the composite. Acoustic and X-ray imaging results were compared to validate the carbon agglomeration.

## 1. Introduction

Polymer nanocomposites have become an object of intense interest since the late 1990s, when various types of low-dimensional carbon nanoforms were synthetized and applied in materials science [1,2,3,4]. The idea of nanocomposites is based on the embedding of different types of low-dimensional nanoparticles into the polymeric matrix volume. Carbon (graphite) 1D and 2D nanoforms—single-wall (SWCNTs) and multi-wall (MWCNTs) carbon nanotubes, graphite nanoplatelets (GNPs) or nanoflakes (GNFs)—as well as silicate (clay) nanoplatelets and platelets formed by other types of layered crystals can be used as nanofillers.

High values of aspect ratio $\;l/d=10^{2}÷10^{3}$, where *l* is lateral size and *d* is nanoparticle thickness, make it possible to obtain a dense packing of nanoparticles. Even for minimal values of the nanofiller content $\;w_{f}\;\sim \;\left(0.01÷2\right)\%$, the mean interparticle distance *s* can be compared with the lateral size *l* of 1D and 2D nanoparticles:  s≤l. This dense packing determines the most attractive feature of nanocomposites, namely the ability to improve their properties with small amounts of nanomaterial additives. This enhancement results from the excellent physical properties of the nanoparticles to be employed—the electrical and heat conductivity of carbon nanoforms and their outstanding elastic and strength properties [5,6]. Nanofiller is a dispersed phase in the structure of a composite material; there are various ways to incorporate its properties into the overall properties of nanocomposites. Substantial electric and heat conductivity of the composite are provided due to the formation of a continuous cluster of conductive carbon nanoparticles. In this case, the polymer matrix acts as a mechanical substrate to keep a stable configuration of nanoparticles. Another allocation of functions between the components arises when it comes to the elastic properties of nanocomposites. Elastic properties have been the subject of a great number of papers and reviews [7,8,9,10,11]. Both constituents, polymer binder and nanocarbon reinforcement, participate in forming the elastic response of nanocomposites. The main part of the applied load is carried by the nanocarbon reinforcing elements; the key role here is played by the 1D and 2D reinforcing elements (or their parts), oriented along the direction of the applied load. The function of the polymer binder is to transfer the load from the region where it is applied to the reinforcing nanoparticles, and to redistribute the load between them. This is achieved through shear stresses at the interface between the matrix and the reinforcement, which is known as the shear lag transfer mechanism [12,13,14,15]. When the nanoparticles do not possess a preferable orientation, the elastic response does not depend on the load direction.

The nanocomposite elastic modulus $E_{c}$ can be assessed by employing the mixture rule for elastic moduli in the frame of the Voigt model for the uniform strain [9,10,11,12,13,14,15,16,17,18]. In this model, the *E_c_* value is estimated as the weighted sum of the elastic moduli $E_{m}$ and $E_{f}$ of the matrix and nanocarbon reinforcement, respectively:

(1) $$E_{c}=\left(1-w_{f}\right)E_{m}+\alpha \cdot w_{f}\cdot E_{f},$$

where $w_{f}$—reinforcement content, α—quotient of reinforcement oriented in the applied load direction [16]. For small reinforcement content $w_{f}\ll 1,$ the polymer nanocomposite modulus $E_{c}$ equals the polymer matrix modulus $E_{m}$ plus the nanocarbon reinforcement contribution $\delta E_{c}=\alpha \cdot w_{f}\cdot E_{f}$. This addition is the product of a high value of the reinforcement modulus $E_{f}$ ($E_{f}/E_{m}\sim 10^{3}$) and α small quantity of the reinforcement content $\left(\alpha \cdot w_{f}\right)$ ($w_{f}\sim 10^{-2}÷10^{-3}$ and α<1). Finally, the reinforcement addition $\delta E_{c}$ is compared with the polymer elastic matrix modulus $E_{m}$:

(2) $$\delta E_{c}=\left(0.1÷1.0\right)E_{m}.$$

The elastic properties of carbon nanocomposites have been extensively studied experimentally, with the results summarized in several publications, including [2,19,20,21,22,23,24,25]. The experiments show a difference in the elastic modulus between the pure polymer and the polymer with carbon nano-dispersions, which gives an enhancement in elastic properties within the range established by Equations (1) and (2). However, some studies show minimal changes in elasticity, with variations in elastic modulus within the confidence interval of experimental error. This lack of enhancement may be due to failure in shear lag transfer. Load transfer requires tight physical contact between the reinforcement and matrix; violations of this contact prevent it. Thus, both the content of the nanofiller and the state of the interface determine the elastic modulus of polymer nanocomposite materials.

An important characteristic of composites is the spatial distribution of their dispersed phase [26,27,28]. The optimal version is a uniform dispersion of nanoparticles, but experimental studies suggest an inhomogeneous distribution of nanocarbon reinforcement. Data from small-angle X-ray (SAXS) and neutron scattering [29,30] in epoxy–carbon composites as well as direct observations of the microstructure on sample cuts using scanning electron microscopy (SEM) [26,27,28] show that the carbon reinforcement is distributed throughout the composite volume in the form of fractal nanoparticle aggregates.

Agglomerates involve many nanotubes, platelets or flakes; the agglomerate size *L* is significantly larger than the lateral size of individual nanoparticles: *L* >> *l*. According to data of the SAXS and SEM images, the value of *L* is in the range of 10–50 µm. Such agglomerates essentially disturb the homogeneity of elastic properties. The local elastic modulus *E*$\left(\vec{r}\right)$ can be estimated from the relation (1) using the local value of reinforcement content $w_{f}\left(\vec{r}\right)$. The separation of nanocarbon reinforcement into agglomerates and its non-uniform distribution throughout the polymer matrix is not essential for large-scale deformations, such as macroscopic loads or low-frequency ultrasonic testing. However, they become critical for high-frequency ultrasound due to its scattering at small-scale elastic inhomogeneities. Scattering efficiency depends on the ratio between the characteristic size of elastic inhomogeneity *L* and the probe ultrasound wavelength *λ*, as well as on the difference between the elastic properties of the perturbed area and the matrix medium.

Ultrasound is an efficient tool for non-destructive observation of the volume structure of composite materials and measurement of their integral and local elastic properties, revealing structural failures within their volume. Different ranges of ultrasonic frequencies provide various levels of spatial resolution and are employed to solve different problems in assessing the structure and mechanical properties of composites. Scanning impulse acoustic microscopy (SIAM), based on applying ultrashort probe pulses of high-frequency (50–200 MHz) focused ultrasound, provides bulk visualization with spatial resolution of 20–100 µm. These values are too large to resolve individual carbon nanoparticles (lateral sizes *l* ~ 1–15 µm) but are enough to reveal regions of increased stiffness resulting from nanoparticle aggregation. One of the most substantial advantages of ultrasonic visualization techniques is their non-destructive nature. In the case of impulse acoustic microscopy, its application makes it possible to see the internal microstructure of a specimen without cutting it.

Papers [31,32,33] describe the use of the SIAM technique for the in situ monitoring of microstructure in nanocomposites containing different types of nanocarbon fillers. Ultrasonic imaging gives unexpected results—acoustic images at different depth inside the nanocomposite specimens include many bright small spots and dots against the dark background. Positions of the spots change when the imaging layer is varying its depth in the specimen volume, but the general character of the images remains unchanged. This means that the bulk microstructure of carbon–epoxy specimens includes many efficient microscopic scatterers. It is unlikely that such scatterers are the agglomerates themselves—magnitudes of the elasticity variations in the regions of carbon nanoparticle agglomeration, as follows from the estimation (1), are not sufficient to provide such effective scattering. The assumption is that the scatterers are air bubbles encapsulating fractal agglomerates within themselves. Such bubbles are formed from air captured by fractal agglomerates during the nanocomposite formation and firmly held by the agglomerates thereafter. Most of the filler nanoparticles are distributed in these voids; they do not contact the polymer matrix and do not participate in the elastic response of the composite material as has been discussed above. This conclusion is corroborated by the results of local elastic measurements performed with the ultrasonic probe beam in different regions of the nanocomposite specimen volume. Local elastic measurements give the same values of sonic velocities at different points of the nanocomposite; these values do not differ from their values in the polymer binder [33].

The formation of such a structure with numerous micro-voids should be explained by the low wettability of carbon filler particles with the polymer binder. It has been shown that the formation of 2D and 3D fractal structures substantially increases the non-wettability of the primary graphite in use for preparing such structures [34]. It can be assumed that assembling nanoparticles into fractal agglomerates and then forming and retaining air cavities around these agglomerates is energetically preferable over direct contact of carbon nanoparticles with the polymer matrix. Apparently, a natural way to eliminate the formation of air bubbles is to prepare carbon nanocomposite samples in a vacuum. However, modifying carbon nanocomposite production using vacuum technologies preserves the overall picture of their internal microstructure with multiple microscopic scatterers [33].

This publication presents experimental data obtained with 3D non-destructive imaging with a sufficient resolution to observe the fine structure of the nanocarbon agglomerates. This is carried out in order to confirm the hypothesis that carbon nanoparticles tend to assemble into loose agglomerates enriched with air that prevents the improvement of the mechanical properties of polymer nanocomposites. The aim of this work is to establish their actual geometry, sizes and interior details. Two high-resolution bulk imaging techniques have been applied. One of them is an enhanced version of the same impulse acoustic microscopy with an essentially increased resolution of 10–20 μm by augmenting the operation frequency up to 200 MHz instead of the 50–100 MHz used in the previous experiments [32]. The alternative technique is X-ray microtomography (X-ray μ-CT) with submicron resolution [35]. The proposed visualizing techniques employ different contrast mechanisms to present the object volume structure but, unlike conventional methods of light, electron or probe microscopy, they make it possible to restore the real three-dimensional distribution of carbon nanofillers even in the case of the formation of fractal agglomerates encapsulated in air micro cavities.

## 2. Materials

Epoxy–carbon nanocomposites were epoxy resin containing small concentration of carbon particles of diverse origins. Epicote 828 epoxy resin (Westlake Chemical Corporation, Houston, TX, USA) with curing agent called A1 (a modified TEPA) was employed as matrix material. Various kinds of small graphite particles were used as fillers. Exfoliated graphite (EG) has a size of 50–200 µm. Graphite nanoplatelets (GNPs) have thickness of 7–10 nm and lateral dimensions of up to 1–10 μm. The latter are multi-walled carbon nanotubes (CNTs). Nanotubes are 20–40 nm in diameter and have a length of 5–10 μm. Sample names and filler concentrations are given in Table 1.

The composite samples were fabricated by solidification of graphite particle suspension in epoxy resin with the curing agent. The fabrication procedure was aimed at achieving the highest homogeneity of specimens [33]. The procedure included (1) vacuum degassing of liquid epoxy resin for 48 h under a few millibars; (2) preparation of graphite nanofiller suspension in isopropyl alcohol and its sonication in ultrasonic bath for 1.5 h; (3) by-hand mixing the liquid resin and graphite particle suspension in isopropyl alcohol; (4) entirely evaporating the alcohol at 150 °C and sonicating for 1.5 h to degas the new mixture; (5) adding curing agent A1 and by-hand mixing it with the graphite–epoxy suspension for 7 min; (6) curing the suspension for 24 h in air at normal conditions and for 4 h in an oven at 80 °C. Samples were cut from prepared plates of composites. Sample dimensions were selected based on the parameters of both imaging methods. So, the size was approximately 0.5 × 0.5 × 5 mm. The two surfaces were plane parallel.

## 3. Methods

Two advanced visualizing techniques have been employed for high-resolution study of the internal microstructure of nanocarbon–epoxy composites.

The impulse acoustic microscopy [36,37] is based on receiving reflected or scattered echo impulses generated by interaction of ultrashort probe pulses of focused high-frequency ultrasound with the volume structure within the focal area of the probe ultrasonic beam penetrated in a specimen. The volume microstructure is recovered by mechanical scanning of the probe ultrasonic beam and time resolving of the recorded signals at each point in the scanning area. The resolution of the technique is defined by the usual Rayleigh expression conventional for all wave technology of visualization.

X-ray computer tomography (CT) is based on measuring the attenuation of the X-ray probe beam as it propagates through a specimen with following mathematical processing and recovering of 3D distribution of the X-ray density of the material [35]. The resolution of the methods is defined by pixel sizes of X-ray detectors used in the tomograph. Structural studies of carbon nanocomposites in our experiment have been performed with synchrotron radiation, which provides submicron resolution imaging with phase contrast enhancement. In [38], a high efficiency of synchrotron radiation microtomography method was shown in the example of reinforced composites based on carbon fibers and epoxy resin.

The scanning impulse acoustic microscope SIAM [39] designed and produced by the Emanuel Institute of Biochemical Physics RAS was used for monitoring of the volume microstructure of nanocomposites. Here, we applied an acoustic lens with a working frequency of 200 MHz and half-aperture of 11°, which has lateral resolution of 24 µm. Short probing pulse of 15 ns duration generated by the electronic block provides depth resolution of 20 µm inside the composites. The acoustic lens is fixed in a precision mechanical scanning system (XYZ-axis) with a scanning step of 5 µm (the reproducibility of lens position is 0.5 µm). During the scanning of the specimen with the lens, the ultrasound beam passes through the water immersion, is reflected from the sample surface and bottom elements of internal structure and is received by the same acoustic lens. The received signals are digitalized by 12-bit ADC at a sampling frequency of 1 GHz (2 × 500 MHz) and averaged up to 8 times at each point of observation. The received echo signal includes time-resolved echoes coming from elements or boundaries located at different depths inside the sample volume. The signals have been saved simultaneously with the scanning coordinates (X-Y coordinates). The stored database is presented as a 3D tomography mode or layer-by-layer images of horizontal cross section (C-scans), and image of vertical section (B-scan) depicts distribution of elements in the volume of material.

To investigate the internal structure of the nanocomposite, we utilized a synchrotron radiation beamline provided with a high-quality monochromatic X-ray beam essential for phase-contrast imaging. Our experiments were conducted at the P14 beamline (DESY PETRA III Hamburg, Germany) [40,41]. The monochromatic X-ray photon flux reached over 10^13^ photons per second within a working region of 0.6 × 1.2 mm^2^, with transverse coherence of approximately 500 μm vertically and 20 μm horizontally. This beamline configuration had previously proven successful for diverse advanced X-ray studies, including X-ray diffraction analysis of microcrystals and large (>100 μm) macromolecular crystals [42,43] and µ-CT of low-density materials [44]. Samples were affixed to a needle holder and exposed to an uncorrected X-ray beam. For tomographic data collection, the holder was rotated with high accuracy (<0.001°). X-ray image projection images were recorded using an optical system comprising an 8 μm thick LSO:Tb scintillator, a 20× microscope and a 2048 × 2048 sCMOS camera (pco.edge 4.2, Excelitas PCO GmbH, Kelheim, Germany). Factoring in the microscope’s magnification, the effective pixel size of the object was estimated to be around 0.325 μm across a field of view of approximately 600 μm. A typical tomographic scan consisted of 30 flat-field images and 3600 projections at intervals of 0.1°. Flat-field correction was implemented by dividing each projection image by the most similar flat field chosen based on the SSIM criterion [45]. To accommodate potential axis shifts at varying object–camera distances, Fourier space correlation with sub-pixel interpolation was employed to correct the X-ray scan sequence images [41]. Phase contrast effect during X-ray projection acquisition was achieved by repetitively scanning the same sample at different distances (135–145 cm) from the camera. Correspondingly, the sets of recorded X-ray projection images underwent processing using a multi-distance non-iterative holographic reconstruction method [46,47]. Following this, tomographic reconstruction was performed using the TOMOPY package [48,49], employing the Gridrec algorithm and Shepp–Logan filtering. For visualization of the reconstructed data in 3D views, we utilized the following image processing software: Fiji-ImageJ 1.53t (open-source) and CTvox v. 3.3.0 r1403 (Bruker MicroCT, Kontich, Belgium).

## 4. Results

**Figure 1:**
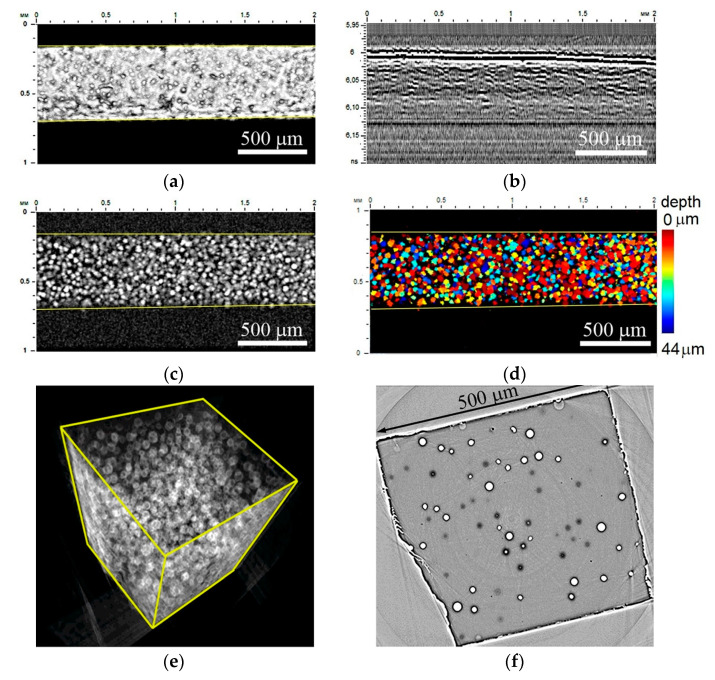
Internal microstructure of epoxy–CNT nanocomposite: (**a**)—acoustic image of surface, (**b**)—B-scan in the central part, (**c**)—C-scan at depth of 40 µm and thickness of 44 µm in classic gray scale gradation and (**d**)—a color distribution of agglomerations over the depth. Working frequency is 200 MHz, scanning field is 1 × 2 mm, scanning step 5 µm. (**e**,**f**)—Three-dimensional rendering and a slice of the sample obtained by X-ray tomography at the PETRA III synchrotron.

**Figure 2:**
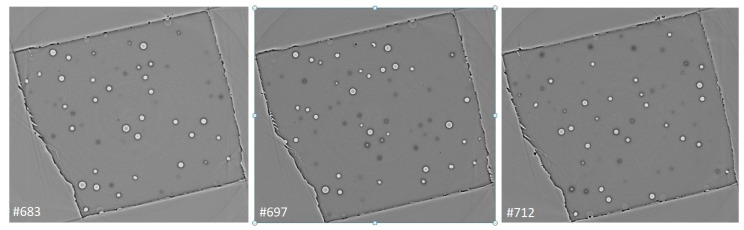
The set of the serial tomographic slices distanced at 10 µm from each other for the epoxy–GNP nanocomposite. Image size was 660 × 660 µm. The thickness of a slice was 0.325 µm.

**Figure 3:**
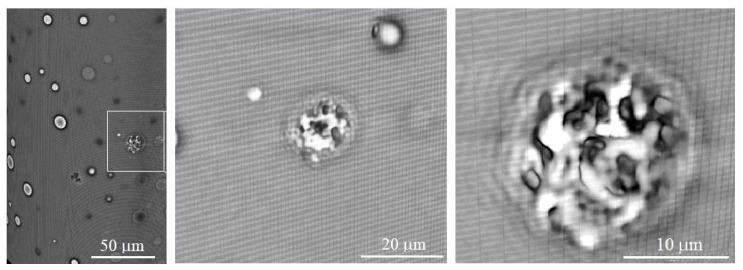
X-ray imaging of CNT cluster structure inside of the individual pore: the bulk composite reconstruction (on the **left**) and high-resolution 2D images of the box (on the **right**).

The results of the visualization of the microstructure in the volume of nanocomposites are presented as ultrasound and X-ray images (Figure 1, Figure 2 and Figure 3). All the obtained images, both acoustic and X-ray, show a characteristic feature of the development of a fractal structure that is the agglomeration of particles with air capture. This is evidenced by the high contrast of the images, which is determined by a large difference in the acoustic properties (impedances) of the epoxy matrix and air in the case of acoustic sensing, and by a significant difference between the absorption of radiation in the matrix material, reinforcing particles and air for X-rays. In addition, the observed inhomogeneities in these images have a regular spherical shape and smooth boundaries.

Figure 1 shows the results of the visualization of the microstructure of a nanocomposite with carbon nanotubes (epoxy–CNT). In the image of the sample surface, the distribution of brightness corresponds to the microrelief caused by the caverns with CNT agglomerates inside (Figure 1a). This relief is a result of the specimen fabrication and the presence of pores in the nanocomposite material. The contrast of the pores was observed due to their sharp edges and scattering of ultrasonic waves at their boundaries. We observe the same pores in the volume as the bright inclusions in the C-scan (Figure 1c) and short bright lines in the B-scan (Figure 1b). These results agree with previous results presented in [31,32,33]. The depth distribution of filler conglomerates is presented using a color scale (Figure 1d). The structure of the composite looks much more interesting in the X-ray image. Figure 1e shows the reconstructed 3D microstructure of the material. One can see multiple points or clusters of minimal X-ray absorption (minimum X-ray density) and their rather compact distribution in the volume. The ideal spherical shape of such inclusions is clearly visible in individual X-ray sections (Figure 1f).

The sizes of visible clusters measured in X-ray images vary in the range of 20–30 µm. In a single slice, the clusters of different sizes are imaged depending on their position with regard to the slice plane. The average distances between the pores depend on the concentration of reinforcing particles. Here, in the slice of thickness 0.35 µm for the concentration 0.25 wt.% of CNTs, the average density of the carbon clusters encapsulated with air is 120 items/mm^2^. In Figure 2, a set of successive slices located at a distance of 10 µm from each other demonstrates the pore configuration dynamics as one moves through the depth of the specimen.

In the X-ray images, we have revealed fairly large pores, and the CNT agglomerates are visible inside the largest of them. Figure 3 shows an image of such an agglomerate in an air capsule with a gradual increasing magnification. It is clearly seen that there is a heterogeneous structure inside the cluster. Dark areas show higher X-ray density and correspond to the carbon nanoparticles; light areas are the air interlayer. Clusters are visible against the background of the polymer matrix.

**Figure 4:**
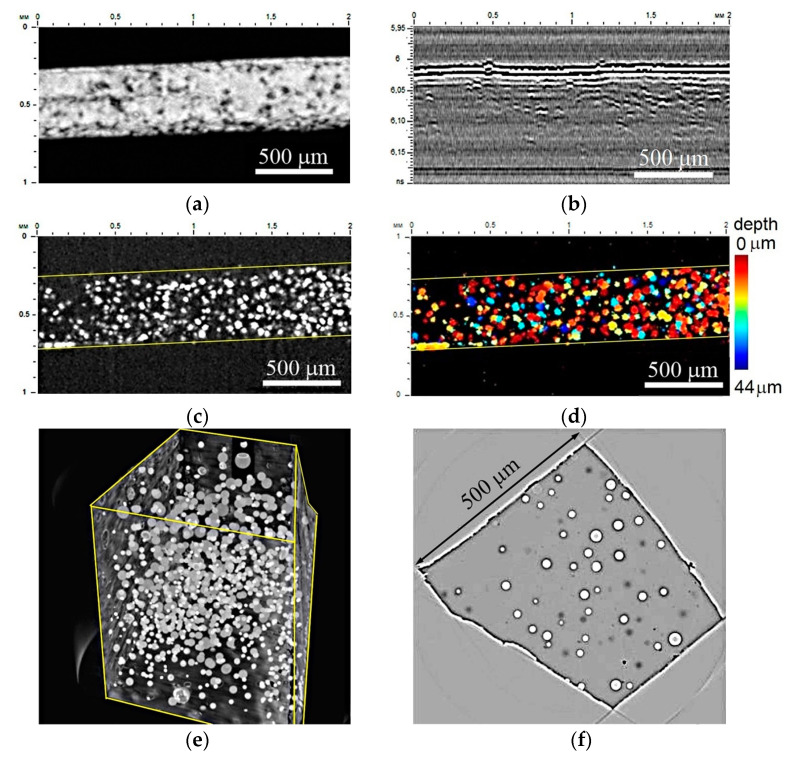
Internal microstructure of epoxy–GNP nanocomposite: (**a**)—acoustic image of surface, (**b**)—B-scan in the central part, (**c**)—C-scan at depth of 40 µm and thickness of 44 µm in classic gray scale gradation and (**d**)—a color distribution of agglomerations over the depth. Working frequency is 200 MHz, scanning field is 1 × 2 mm, scanning step is 5 µm. (**e**,**f**)—Three-dimensional rendering and a slice of the sample obtained by X-ray tomography at the PETRA III synchrotron.

Figure 4 shows the GNP composite microstructure and distribution of graphite particles in the volume of the nanocomposite. In general, the observed microstructure looks the same as that of the CNT composite; that is, it is an agglomeration of the carbon particles encapsulated with air into pores. The pores have different sizes in the range from 10 up to 50 µm. Their distribution is dense; the average density of the pores and carbon clusters is increased up to 200 items/mm^2^. The heterogeneity of pore distribution is revealed by acoustic images (Figure 4a–d). In Figure 4c, the integral number of pores is visible in the layer of 44 µm thickness with a central depth of 40 µm. The colored image (Figure 4d) gives the distribution of pores at a certain depth; it is easy to reveal that the pore sizes vary at the same depth. Figure 4e,f present a 3D reconstruction of the GNP composite specimen volume and its microstructure in an individual slice of 0.35 µm. The tomographic representation allows for seeing that the brightness of the pores varies; some of the largest pores have a GNP agglomerate structure inside, similar to that presented for the CNT composite in Figure 3. Individual GNP particles were not identified in tomographic images.

**Figure 5:**
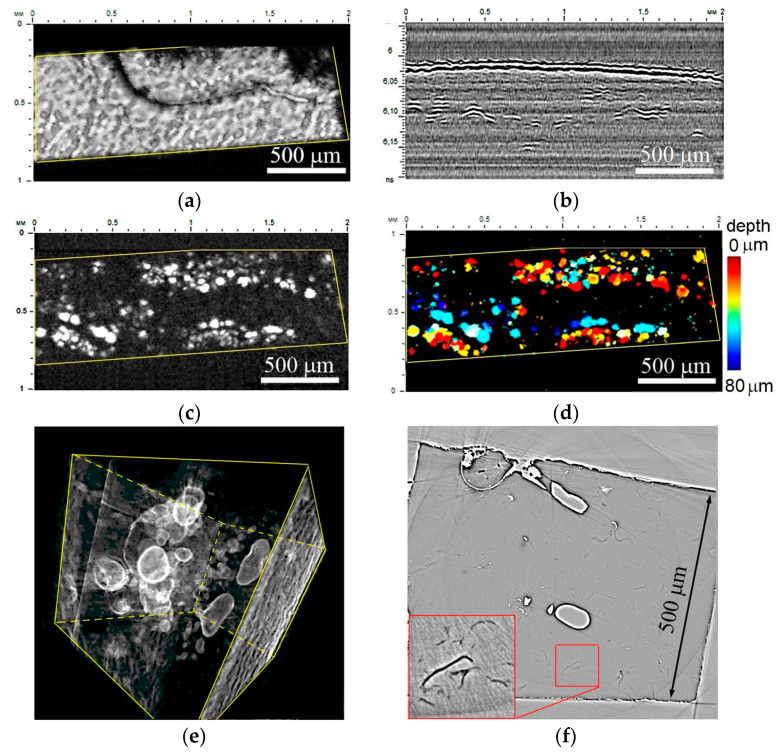
Internal microstructure of epoxy–EG nanocomposite: (**a**)—acoustic image of surface, (**b**)—B-scan in the central part, (**c**)—C-scan of the layer at depth of 40 µm and 80 µm thickness in classic gray scale gradation, (**d**)—a color distribution of agglomerations over the depth. Scanning field is 0.8 × 2 mm, scanning step 5 µm. (**e**,**f**)—Three-dimensional rendering and a slice of the sample obtained by X-ray tomography at the PETRA III synchrotron. In (**f**), the red box shows an enlarged defect structure.

Investigation of the EG composite samples demonstrates another microstructure formation (Figure 5). A distinctive feature of this microstructure is the presence of large inclusions. The inclusions are extended (≈150 µm) and have complex rounded non-spherical shape with smooth borders, as can be seen on tomographic images (Figure 5e,f). The core of these inclusions is EG particles and the boundaries are formed depending on the degree of wettability of the particles with the polymer matrix (epoxy resin). The shape of the observed inclusions corresponds to the EG particles shape, and there is an enhanced contrast of the particle borders caused by the air presence around the particle.

Acoustic images (Figure 5b–d) show only the top parts of the curved surface of the inclusions, the normal to which lies inside the aperture of the receiving lens. Due to this specificity of the acoustic image formation of curved surfaces, we can only detect the small bright spots (50–100 µm).

Cracks in the polymer matrix were observed for these types of samples; the largest one was visible on the acoustic image of the surface that expanded from the surface deep into the sample (Figure 5a). The X-ray slice demonstrates the distribution of cracks in the sample body (Figure 5f).

## 5. Discussion

The use of two effective high-resolution imaging methods makes it possible to obtain high-contrast, detailed 3D images of the volumetric microstructure of carbon–epoxy nanocomposites. In scanning pulsed acoustic microscopy, images are formed by echo pulses reflected from interfaces or backscattered by small inclusions; they represent the distribution of such interfaces or inclusions over the volume of the sample. The contrast of the images is determined by the difference in acoustic properties at the boundaries, the maximum value of which is reached at the face with air or a vacuum. As opposed to the AM method, X-ray tomographic images are obtained in phase-contrast-enhanced transmission mode. In this case, the distribution of X-ray absorption over the sample bulk is displayed; the maximum X-ray contrast occurs for areas with finite X-ray density—normal to the material—and areas with zero absorption—air or a vacuum. In our experiments with both methods, we observed high contrast, so it is natural to assume the presence of air in the area of agglomeration of the reinforcing particles. Encapsulation of graphite particles (agglomerates) excludes the participation of filler in the elastic response of the material to an external mechanical load.

A fairly large number of publications are devoted to the study of elastic properties of nanocomposites [19,20,21,22,23,24,25]. The study of the dependence of the composite modulus on the concentration of the reinforcing additive is one of the main tasks of these works. In accordance with Formula (1), an increase in the content of reinforcing particles should increase the elastic modulus. In the cited references, a completely different nature of this dependence was observed [19,20,21,22]. Some of them report a decrease in the modulus in a polymer with a higher concentration of carbon filler. In our previous work [31,32,33], which was performed with nanocomposite samples manufactured using the same technique as in this article, the change in elastic moduli at different filler concentrations was within the confidence interval. These data are consistent with the conclusions of this work that reinforcing elements can be minimally embedded in the matrix material.

The results obtained in this work indicate that the wettability of reinforcing particles is a key factor for improving the elastic properties of the nanocomposite. Complete wettability is possible when all reinforcing components are involved in the formation of the elastic response; in the absence of wettability, when reinforcing elements are encapsulated with air, even a decrease in the elastic modulus is possible. In the intermediate case, when there is partial embedding of the forming particles into the polymer matrix, some enhancement in elastic moduli may be observed, depending on the concentration of particles successfully merged into the polymer.

## 6. Conclusions

Two effective high-resolution non-destructive imaging techniques were applied for detailed imaging of the 3D bulk structure of carbon–epoxy nanocomposites.

It is shown that nanocomposites obtained by standard methods from GNPs and multi-walled CNTs without additional modification of the particle surface result in a composite microstructure filled with encapsulated fractal agglomerates of carbon particles, which can be impeding for the elastic property enhancement.

Due to the high resolution of the synchrotron X-ray technology used in this work, it was possible to observe the image of thin slices and identify the structure of individual agglomerates. It can be seen that the agglomerate is a collection of carbon nanoparticles that capture a certain amount of air during material preparation and have an insufficiently active surface to ensure full adhesion of the filler to the polymer matrix. This situation leads to a lack of improvement in the elastic properties of the composite compared to a pure polymer, since the condition of the continuity of mechanical stresses is violated at the local boundaries of nanoparticle agglomerates, and the particles do not contribute to the elastic response of the material as a whole.

The high-resolution X-ray method is quite time-consuming, expensive and applicable for specially prepared samples. The results of this study demonstrate that acoustic microscopy is a highly sensitive express technique for detecting air cavities. This is the reason for using the technique to evaluate the effect of reinforcement particle agglomeration and air encapsulation on the reinforcement as well as to estimate the mechanical properties of nanocomposites materials.

## Figures and Tables

**Table 1:** Legend of samples.

Sample	Filler	Content, wt.%	Nanoparticle Sizes	
			Thickness *d*	Lateral Size *l*
epoxy–EG	exfoliated graphite	0.25	50–200 nm	50–200 nm
epoxy–GNPs	graphite nanoplatelets	0.5	≈10 nm	1–10 μm
epoxy–CNTs	carbon nanotubes	0.1	Ø 20–40 nm	5–10 μm

## Data Availability

Data are contained within the article.
